# Primary Indicators to Systematically Monitor COVID-19 Mitigation and Response — Kentucky, May 19–July 15, 2020

**DOI:** 10.15585/mmwr.mm6934e3

**Published:** 2020-08-28

**Authors:** Kate Varela, Benjamin Scott, John Prather, Erin Blau, Peter Rock, Adam Vaughan, Cara Halldin, Sean Griffing, Heidi Pfeiffer, Janine Hines, Emilio Dirlikov, Doug Thoroughman

**Affiliations:** ^1^Epidemic Intelligence Service, CDC; ^2^Kentucky Department for Public Health; ^3^Kentucky Injury Prevention and Research Center, Lexington, Kentucky; ^4^Center for Clinical and Translational Science, University of Kentucky, Lexington, Kentucky; ^5^CDC COVID-19 Response Team; ^6^Career Epidemiology Field Officer Program, CDC.

*On August 25, 2020, this report was posted online as an *MMWR *Early Release.*

State and local health departments in the United States are using various indicators to identify differences in rates of reported coronavirus disease 2019 (COVID-19) and severe COVID-19 outcomes, including hospitalizations and deaths. To inform mitigation efforts, on May 19, 2020, the Kentucky Department for Public Health (KDPH) implemented a reporting system to monitor five indicators of state-level COVID-19 status to assess the ability to safely reopen: 1) composite syndromic surveillance data, 2) the number of new COVID-19 cases,* 3) the number of COVID-19–associated deaths,^†^ 4) health care capacity data, and 5) public health capacity for contact tracing (contact tracing capacity). Using standardized methods, KDPH compiles an indicator monitoring report (IMR) to provide daily analysis of these five indicators, which are combined with publicly available data into a user-friendly composite status that KDPH and local policy makers use to assess state-level COVID-19 hazard status. During May 19–July 15, 2020, Kentucky reported 12,742 COVID-19 cases, and 299 COVID-19–related deaths (*1*). The mean composite state-level hazard status during May 19–July 15 was 2.5 (fair to moderate). IMR review led to county-level hotspot identification (identification of counties meeting criteria for temporal increases in number of cases and incidence) and facilitated collaboration among KDPH and local authorities on decisions regarding mitigation efforts. Kentucky’s IMR might easily be adopted by state and local health departments in other jurisdictions to guide decision-making for COVID-19 mitigation, response, and reopening.

On March 6, Kentucky reported its first COVID-19 case and declared a state of emergency. During subsequent weeks, mitigation efforts included temporarily closing schools for in-person instruction, ceasing elective medical procedures, and limiting visitors to long-term care facilities; an executive order was issued on March 22 that temporarily closed all nonessential businesses. The number of cases during March 6–May 8 peaked during the week of May 4, when 1,446 cases were reported (*1*). Kentucky commenced reopening on May 9 through the phased “Healthy at Work” plan.^§^ During reopening, KDPH and other officials sought to monitor changes in rates of reported COVID-19 and health care resource utilization to inform mitigation and reopening policies (*2*). KDPH epidemiologists developed the IMR after recognizing the need for a plain language assessment that could facilitate reopening and ongoing response decision-making addressing multiple stakeholders. The five primary indicators were selected based on available data and in consultation with KDPH syndromic surveillance and emergency preparedness subject matter experts and academic advice from the University of Kentucky and the Kentucky Injury Prevention and Research Center. Metrics were developed in consultation with CDC COVID-19 Response task force modeling experts. KDPH implemented the IMR process on May 19. The IMR describes five state-level primary indicators (syndromic surveillance data, case counts, deaths, health care capacity data, and contact tracing capacity), which are scored individually. Scores are combined into a composite categorical state-level status indicator to assess COVID-19 disease prevalence and severity (syndromic surveillance data, cases, deaths) and readiness (health care capacity and contact tracing capacity). Daily IMRs are standardized and produced with publicly available data (*3*) using spreadsheets and R statistical software (version 3.6.3; The R Foundation). Reports are produced and results are disseminated Monday through Saturday. Reports include data through the report date.^¶^

The slope of the 7-day moving average for seven separate variables constituted the indicator for syndromic surveillance data (*4*). These state-level variables were inpatient admissions, outpatient visits, and emergency department (ED) visits attributed to COVID-19–like illness (variables 1–3); inpatient admissions, outpatient visits, and ED visits attributed to COVID-19 diagnostic codes (variables 4–6); and ED visits attributed to influenza-like illness (variable 7).

The case count indicator was assessed as a composite of the number of new COVID-19 cases per 100,000 population reported to KDPH during the preceding 2 weeks (incidence) and the slope of the 7-day moving average (incidence trend). State-level incidence was categorized as low (≤10 per 100,000 population), moderate (>10–49.99), moderately high (≥50–100), and high (>100). The slope of the 7-day moving average was categorized as decreasing (≥4 days with slope <0) or increasing (≥4 days with slope ≥0).

Similarly, the COVID-19–associated death indicator was a composite of COVID-19-associated mortality per 100,000 in the preceding 2 weeks and the slope of the 7-day moving average. The state-level mortality rate was categorized as low (≤1.5 per 100,000), moderate (>1.5–2.99), moderately high (≥3–5), and high (>5). As with cases, the slope of the 7-day moving average was categorized as decreasing (≥4 days with slope <0) or increasing (≥4 days with slope ≥0).

The health care capacity indicator was a composite measure that included 1) state-level hospital utilization as the percentage of intensive care unit beds in use and the percentage of ventilators in use as reported daily by Kentucky health care facilities to WebEOC (https://www.juvare.com/webeoc/), an emergency management software application used by the KDPH Public Health Preparedness Branch, and 2) the supply of personal protective equipment as measured by state-level N95 respirator availability, which is based on information collected by KDPH in a state-level supply database. Finally, the contact tracing capacity indicator was measured as the daily percentage of contact tracing teams deployed to each of the 16 public health regions in Kentucky.

Each of the five indicators was scored using a 3-point scale (3 = excellent, 2 = moderate, 1 = poor) (Supplementary Table, https://stacks.cdc.gov/view/cdc/91982). A daily state-level composite COVID-19 status was determined by the number of individual indicators that were excellent. Each indicator was weighted equally and accounted for 20% of the composite status. This daily composite COVID-19 status was described by a user-friendly, descending 5-point rating system developed around reopening recommendations (5 = excellent [reopen/remain open]; 4 = good [monitor, continue reopening/remain open], 3 = moderate [caution, enhance monitoring], 2 = fair [increase mitigation], 1 or 0 = poor [reopening risky, slow reopening or close]). The daily IMR included the five indicators, the composite state-level COVID-19 status, and data to support the score for each indicator. County-level incidence hotspot maps were compiled in the IMR to help focus investigation efforts on hotspots as they were identified.

The mean scores for each indicator during May 19–July 15, 2020, were calculated by summing the products of the scores multiplied by the number of days with that score and dividing by the total number of days assessed. The same method was used to calculate means for the IMR composite COVID-19 status.

KDPH reported 12,742 incident COVID-19 cases and 299 COVID-19–related deaths during May 19–July 15, 2020; 5,705 (44.8%) cases occurred in males, and the median age was 41 years (range = 0–107 years). During this period, the mean COVID-19 status was 2.5 (fair to moderate) (range = 2–4) (Figure). The composite status was 4 (good) for 19 days (38.7%) and 3 (moderate) for 22 days (44.8%). Eight days were rated as 2 (fair); five of these occurred after June 29. No days were rated as 5 (excellent), 1 (poor), or 0 (poor). During May 19–June 16, the mean state-level composite status was 3 (moderate); during June 17–July 15, the mean composite status was 2.5 (fair to moderate).

**FIGURE:**
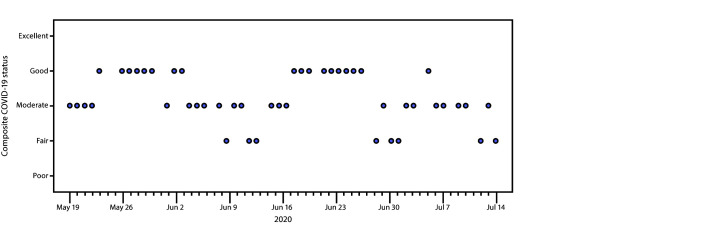
State-level composite COVID-19 status*^,^^†^— Kentucky, May 19–July 15, 2020**^§^** **Abbreviation:** COVID-19 = coronavirus disease 2019. * Kentucky’s state-level composite COVID-19 status assesses the ability to safely reopen and remain open. COVID-19 status was reported at five levels: 5 = excellent (reopen/remain open); 4 = good (monitor); 3 = moderate (caution); 2 = fair (increase mitigation); 1 = poor (reopening risky, slow reopening or close); 0 = poor (reopening risky, slow reopening or close). ^†^ COVID-19 status is based on indicator monitoring reports (IMRs), which are produced daily by the Kentucky Department of Public Health, Monday through Saturday, and include data through the report date. The five indicators used to generate the composite COVID-19 status include 1) syndromic surveillance data; 2) the number of new COVID-19 cases; 3) the number of COVID-19–associated deaths; 4) health care capacity data; and 5) public health capacity for contact tracing. No data are reported on Sundays. The Monday IMR includes cumulative Sunday cases, deaths, and syndromic surveillance data. Sunday contact tracing capacity and health care capacity data were not reported. ^§^ No IMR was produced on May 25 because of the Memorial Day holiday; the May 26 IMR included May 25 data.

During May 19–July 15, 2020, the mean score for syndromic surveillance data was 2.0 (moderate) (range = 1–3), with 20 consecutive days of excellent during May 19–June 12, followed by periods of nonconsecutive days where the score was excellent (17 days), moderate (6 days), and poor (6 days), with scores of poor on three consecutive days during July 13–July 15 (Table). The mean score for the case count indicator was 2.5 (poor to moderate) (range = 1–3), with scores of poor on 22 consecutive days from June 20 to July 15. Mean death indicator was 2.5 (moderate to excellent) (range = 2–3). Death indicator score changes most frequently resulted in a change in the composite COVID-19 status (13 instances). Mean health care capacity was 3.0 (excellent) (range = 3), remaining unchanged throughout the period. Mean contact tracing capacity was 2.0 (moderate) (range = 1–3). As of June 2, contact tracing capacity increased from 0% to 100% when all 16 Regional Epi Contact Tracing Teams were deployed to assigned regions and available to conduct case and contact investigations.

**TABLE:** COVID-19 hazard status indicator score results, based on indicator monitoring reports* — Kentucky, May 19–July 15, 2020

Indicator	No. of days with score†			Average daily score	No. of times status changed because score changed	Max. no. of days^§^ with poor score (date range)
	Excellent	Moderate	Poor			
Syndromic surveillance data	37	6	6	2.0	6	3 (Jul 13–Jul 15)
COVID-19 cases	5	13	31	1.5	6	22 (Jun 20–Jul 15)
Associated deaths	29	20	0	2.5	13	0 (—)
Health care capacity	49	0	0	3.0^¶^	0	0 (—)
Public health capacity for contact tracing	37	1	11	2.0	3	11 (May 19–Jun 1)

**Abbreviation:** COVID-19 = coronavirus disease 2019.

* Indicator monitoring reports compiled by the Kentucky Department of Public Health.

^†^ Excellent = score of 3; moderate = score of 2; poor = score of 1.

^§^ Days were consecutive.

^¶^ The average daily score for health care capacity remained unchanged (score = 3) during May 19–July 15, 2020.

## Selected Example of IMR Use

On July 7, 2020, the COVID-19 status score in Kentucky was 3 (moderate), prompting additional review of county-level incidence rate maps included in the IMR by KDPH epidemiologists. A suspected hotspot (defined by KDPH as a county with a 7-day average daily incidence rate of >25 cases per 100,000 population) was identified in Bell County, a county that had had a low incidence until that time. The state epidemiologist contacted the regional epidemiologist to confirm that case investigations were underway. Case investigations revealed four specific clusters but did not indicate increased community transmission. The regional epidemiologist reported that appropriate contact tracing and quarantine measures had occurred within 12 hours of notification, and, because additional state-level public health action was not warranted, resources could be directed elsewhere.

## Discussion

Kentucky’s IMR and composite state-level COVID-19 status scores were produced to facilitate decisions regarding reopening and ongoing COVID-19 response decision-making among various stakeholders. The IMR is a tool that combines multiple data elements to systematically assess reopening efforts in the state as measured by a daily composite state-level status score. Kentucky’s COVID-19 status is reported Monday through Saturday to approximately 90 stakeholders within and outside state government, including the Kentucky Governor’s Office and local health department directors. Officials reported monitoring the status daily as a plain language summary of multiple critical indicators to describe the current COVID-19 hazard status in Kentucky. Local health departments also reported COVID-19 status monitoring to track statewide status and maintain vigilance for worsening conditions to inform their local decision-making. Reports such as the IMR, geared toward a broader audience of decision-makers, are important tools for informing and guiding public health policy as the COVID-19 pandemic continues.

During May 19–July 15, the Kentucky composite COVID-19 status worsened. During this period, the COVID-19 status was 3 (good: recommend monitoring) or 2 (moderate: recommend caution) 83% of the time. In certain instances, the composite COVID-19 status was moderate or good despite increasing incidence, which was attributed to all indicators receiving equal weight in the composite status scoring system. However, more recent IMR data indicate declining ratings, with the majority of days having a status of fair (fair: recommend increased mitigation efforts) occurring during June 17–July 15. In Kentucky, incidence has continued to increase, death rates have fluctuated, and syndromic surveillance data have demonstrated increases in ED visits and hospitalizations attributed to COVID-19–like illness and COVID-19. These results are consistent with identified hotspot counties and regions and increasing transmission statewide (*1*). Timely dissemination of easily understood surveillance data are critical to a rapid and effective public health response (*5*). The IMR has supported implementation of mitigation efforts to reduce transmission, including the July 9, 2020, executive order mandating face coverings in certain settings.**

The findings in this report are subject to at least five limitations. First, changes in data reporting or health care utilization might influence interpretation of the five indicators (e.g., increased use of telehealth) (*6*). Second, health care capacity might be affected by unaccounted factors such as the number of patients per nurse in intensive care units. Third, after implementation of the IMR, modifications were made to improve the scoring methods for cases, deaths, and syndromic surveillance data, which might affect comparability over time. Fourth, additional updates might be needed, including a more detailed assessment of levels for contact tracing capacity^††^that includes turnaround time for test results or additional indicators, as response needs change. Finally, because the composite score was derived in consultation with multiple subject matter experts across disciplines, a field assessment is needed to validate the scoring system.

Jurisdictions such as state and local health departments might benefit from use of IMRs to guide decision-making for continued COVID-19 mitigation and response. Data sources included in Kentucky’s IMR are publicly available, data are analyzed with familiar software, and a standardized method is used to compile the report, suggesting IMR might easily be adopted by other jurisdictions.

**Summary**

**What is already known about this topic?**

State and local health departments use various indicators to identify local and regional changes in the number of COVID-19 cases and severe outcomes, including hospitalizations and deaths.

**What is added by this report?**

Kentucky’s indicator monitoring report (IMR) is a useful tool that combines multiple data elements to generate a daily COVID-19 status score that allows systematic assessment of the state’s mitigation, response, and reopening efforts. The Kentucky Department for Public Health analyzes publicly available data sources and compiles the IMR using standardized methods.

**What are the implications for public health practice?**

State and local health departments in other jurisdictions might benefit from implementation of systematic indicator monitoring to guide decision-making for COVID-19 reopening, mitigation, and response efforts.

## Footnotes

* Reported cases include all laboratory-confirmed and probable COVID-19 cases reported to the Kentucky Department for Public Health, using the Council of State and Territorial Epidemiologists case definition. https://cdn.ymaws.com/www.cste.org/resource/resmgr/2020ps/Interim-20-ID-01_COVID-19.pdf.

† A COVID-19 death was defined as any death determined to be caused directly by COVID-19 or for which COVID-19 was listed as a contributing cause on the death certificate. COVID-19 deaths include deaths with or without laboratory confirmation if the decedent met the CSTE probable case definition or through autopsy or epidemiologic findings of the coroner's investigation. Death certificates are examined by the COVID-19 Mortality Review Team on a weekly basis to determine if COVID-19 is listed as a primary or contributing cause of death. For any cases in question, medical records are obtained, and the case is adjudicated by the COVID-19 Mortality Review Team.

§ https://governor.ky.gov/covid19.

¶ Monday IMR included cumulative Sunday cases, deaths, and syndromic surveillance data. Sunday contact tracing capacity and health care capacity data were not reported.

** https://chfs.ky.gov/agencies/dph/covid19/FAQsFaceCoverings.pdf.

†† A more detailed assessment for contact tracing capacity might include percentage of case and contact investigations that occur within a recommended time period with the current number of contact tracers deployed to each of the 16 public health regions of Kentucky and the current incidence in each region.
